# Fetal sex shapes maternal immune adaptation: placental extracellular vesicles differentially reprogram the phenotype, metabolism, and function of circulating monocytes

**DOI:** 10.3389/fimmu.2026.1855111

**Published:** 2026-07-17

**Authors:** Julieta Avalos, Florencia Sabbione, Daiana Rios, Daniel H. Grasso, M. Noe Garcia, Franco Aguilera, Fatima Merech, Horacio Aiello, Cesar Meller, Rosanna Ramhorst, Vanesa Hauk, Soledad Gori, Claudia Pérez Leirós, Daiana M. Vota, Daniel E. Paparini

**Affiliations:** 1 Universidad de Buenos Aires (UBA), Consejo Nacional de Investigaciones Científicas y Técnicas (CONICET), Instituto de Química Biológica de la Facultad de Ciencias Exactas y Naturales (IQUIBICEN-CONICET), Laboratory of Immunopharmacology, Facultad de Ciencias Exactas y Naturales (FCEN-UBA), Buenos Aires, Argentina; 2 Universidad de Buenos Aires, Facultad de Ciencias Exactas y Naturales (FCEN-UBA). Departamento de Química Biológica, Buenos Aires, Argentina; 3 Instituto de medicina experimental (IMEX)-CONICET/Academia Nacional de Medicina de Buenos Aires, Buenos Aires, Argentina; 4 Universidad de Buenos Aires (UBA), Consejo Nacional de Investigaciones Científicas y Técnicas (CONICET), Instituto de Química Biológica de la Facultad de Ciencias Exactas y Naturales (IQUIBICEN-CONICET). Laboratory of Immunomodulation, Metabolism and Cell communication, Facultad de Ciencias Exactas y Naturales (FCEN-UBA), Buenos Aires, Argentina; 5 Department of Biological Sciences, Faculty of Pharmacy and Biochemistry, University of Buenos Aires, Buenos Aires, Argentina; 6 Institute of Humoral Immunity Studies (IDEHU), CONICET, Faculty of Pharmacy and Biochemistry, University of Buenos Aires, Buenos Aires, Argentina; 7 Chair of Immunology, Department of Microbiology, Immunology, Biotechnology and Genetics, Faculty of Pharmacy and Biochemistry, University of Buenos Aires, Buenos Aires, Argentina; 8 Hospital Italiano de Buenos Aires, Servicio de Obstetricia, Buenos Aires, Argentina

**Keywords:** extracellular vesicles, immunometabolism, macrophages, monocyte, pregnancy, sex-differences

## Abstract

**Introduction:**

Maternal immune adaptation during pregnancy is orchestrated by dynamic signals from the uterine microenvironment, including placental extracellular vesicles (pEVs) released into maternal circulation. EVs have emerged as key mediators of this crosstalk; however, their role in sex-specific immune modulation remains incompletely defined. Here, we investigated whether pEVs derived from term placentas induce sex-dependent changes in the phenotype, metabolism, and function of human monocytes.

**Methods:**

pEVs were isolated from 13 term uncomplicated placentas (six male-derived, M-pEVs, and seven female-derived, F-pEVs) and characterized by complementary approaches, revealing similar size distributions and concentrations, with differences in physicochemical properties and molecular cargo. Circulating monocytes from 17 non-pregnant female donors were exposed to M-pEVs or F-pEVs and analyzed for phenotypic, metabolic, and functional responses.

**Results:**

pEVs induced distinct activation profiles depending on fetal sex. F-pEVs reduced CD11b and CD11c expression while increasing CD14, CD39 and IL-10 production. On the other hand, M-pEVs increased CD14 expression and enhanced IL-1β secretion. Both nanovesicles populations increased IL-10 and CXCL8 release and promoted a shift toward classical monocytes (CD14+CD16-) with a reduction in the intermediate subsets. Metabolic analyses revealed divergent immunometabolic programs: M-pEVs promoted lactate and reactive oxygen species production, whereas F-pEVs enhanced lactate production, fatty acid uptake, lipid droplet accumulation, and mitochondrial activity without increasing ROS. Functionally, both pEV populations increased efferocytosis, with a distinct sensitivity to metabolic inhibitors.

**Discussion:**

These findings demonstrate that pEVs differentially modulate circulating monocytes according to fetal sex and support a role for fetal sex in shaping maternal immunometabolic responses.

## Graphical Abstract

**Figure:**
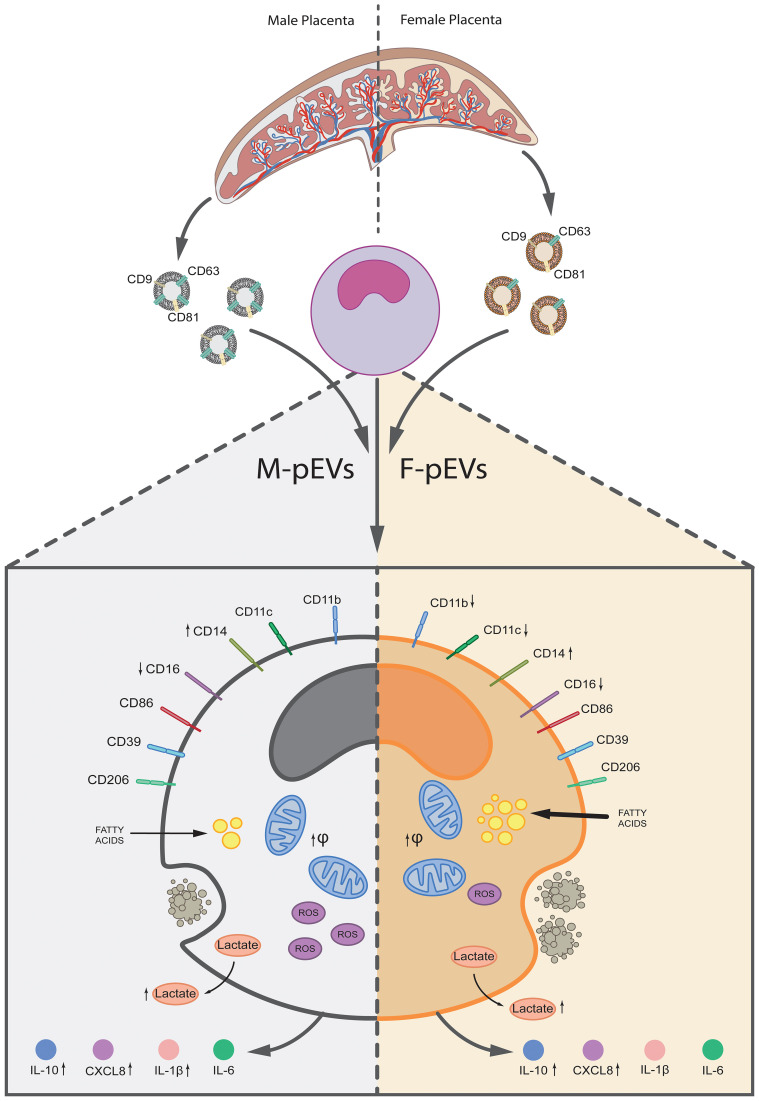

## Introduction

A successful pregnancy requires a finely tuned maternal immune adaptation driven by dynamic communication between the placenta and circulating immune cells (1–3). Extracellular vesicles (EVs) have emerged as key systemic mediators of this crosstalk. Placental extracellular vesicles (pEVs) are released into the maternal bloodstream (4, 5), carrying bioactive cargo (6, 7). Their abundance and composition are dynamically regulated during pregnancy (8) and in pathological conditions such as preeclampsia and fetal growth restriction (9–11) supporting their role as active modulators of maternal physiology. As components of the placental microenvironment, pEVs represent a systemic extension of uterine immune signaling into the maternal circulation.

An emerging aspect of maternal-fetal communication is how fetal sex influences maternal physiology (12–16). Clinical and experimental evidence indicates that fetal sex shapes maternal immune responses, with male pregnancies generally associated with a heightened pro-inflammatory profile (16, 17) and increased susceptibility to certain pregnancy complications, including preeclampsia (18–20). Despite these observations, the mechanisms underlying this sexual dimorphism remain poorly understood.

pEVs are critical in modulating maternal immune cells to support placental development and fetal growth (21–23). Monocytes (Mo) play a central role in maternal immune regulation due to their ability to differentiate into macrophages (MA), produce inflammatory mediators, and participate in tissue remodeling and the clearance of apoptotic cells (24–26). Monocytes and macrophages are highly adaptable cells whose functional phenotypes are closely linked to their metabolic state. Rather than merely supporting cellular activity, pathways such as glycolysis, oxidative phosphorylation, and fatty acid oxidation act as integral regulators that shape the inflammatory and pro-resolving responses in early pregnancy (26, 27) and at term (28). Additionally, recent studies have reported sex-associated differences in the metabolism of placental macrophages (HBCs) (17) and the chorionic villi (17, 29) during pregnancy. Whether pEVs differentially reprogram monocyte phenotype and metabolism according to fetal sex remains largely unexplored.

Here, we provide evidence to support that human placental extracellular vesicles-derived from male and female fetuses (M- and F-, respectively) differentially reprogram circulating monocytes at the phenotypic, metabolic and functional levels. M- and F-pEVs induce distinct metabolic and activation profiles, characterized by differences in ROS production and lipid-associated parameters. These differences are associated with variations in sensitivity to metabolic inhibition of efferocytosis, supporting a role for pEVs in shaping sex-specifiic immunometabolic responses.

## 2 Materials and methods

### 2.1 Samples

Term placental tissue (N = 13, six from male and seven from female placentas, according to the sex of the singleton born) with a gestational age of 39.1 ± 0.6 was collected from women undergoing a scheduled C-section. The women participating in the study were between 26 to 41 years old (33.2 ± 0.3) and had a normal BMI (24.1 ± 0.4). They had negative serology for HIV, HCV, Chagas disease, and syphilis, and had no clinical records of gestational diseases or more than one pregnancy loss. Samples from women with serious complications during pregnancy such as infections that compromised the pregnancy or fetal death, were excluded. Pregnancies with fetal organ malformations or congenital disorders were also excluded. Approval was obtained from the Research Ethics Committee of the Hospital Italiano of Buenos Aires (HIBA 1681). Written informed consent was obtained from all women undergoing elective C-sections. Term placentas were used to culture placental villi explants to obtain extracellular vesicles (EVs).

Monocytes from healthy female donors’ peripheral blood (N = 17) were isolated using Ficoll-Hypaque and Percoll gradient, as previously described (24). The mononuclear cells were used for phenotypic, metabolic and functional assays, cultured either without a stimulus (control, -) or with extracellular vesicles from male (M-pEVs) and female (F-pEVs) placentas for 20 h. Peripheral blood samples from non-pregnant volunteers were obtained as approved by the Argentine Society of Clinical Investigation (SAIC 10469/25). Written informed consent was obtained from all participants prior to inclusion.

### 2.2 Placental extracellular vesicles isolation

Placental villi immediately beneath the *decidua basalis* were dissected as previously described (25, 30). Briefly, small villi explants of a similar size (5 x 5 x 1 mm) and weight were excised from term placenta’s cotyledons, washed with cold PBS, and cultured in 24-well polystyrene plates in 1000 µL of DMEM:F12 supplemented with 2% (v/v) FCS, which was depleted of extracellular vesicles (EVdFCS) by ultracentrifugation (31), and supplemented with 100 µg/mL penicillin/100 U/mL streptomycin (Life Technologies, Buenos Aires, Argentina) for 20 h. The explants were collected in RIPA buffer for protein assays and stored at -80 °C until use. Placental supernatants (SN) underwent differential centrifugation to obtain small extracellular vesicles (pEVs) with the traditional protocol approved by MISEV 2023. Briefly, the culture samples were centrifuged at 2,000× g for 10 min at 4 °C to remove apoptotic cells and debris. Then, SN were centrifuged for 30 min to 10,000 g at 4 °C. Subsequently, ultracentrifugation was performed in the soluble fraction for 70 min at 100,000× g at 4 °C. The pellet was resuspended in PBS, filtered through a 0.22 µm filter (GE Healthcare Life Sciences, Boston, MA, USA) for sterilization and enrichment of small EVs, and then subjected to a second ultracentrifugation. After resuspension, EVs were frozen at −80 °C. The protein concentration was quantified using the Pierce bicinchoninic acid (BCA) protein assay kit. EV purity was estimated by calculating the particle-to-protein ratio obtained from nanoparticle tracking analysis (NTA) and micro-BCA protein quantification. The particle-to-protein ratios ranged from 1.26 to 2.32 × 10^9^ particles per µg of protein, consistent with the MISEV guidelines for moderate-purity extracellular vesicle preparations. RNA was quantified via UV absorbance using a NanoDrop 2,000/2,000c (Thermo Fisher Scientific, Waltham, MA, USA). pEVs characterization was performed according to the Minimal Information for Studies of Extracellular Vesicles (MISEV2023) guidelines from the International Society for Extracellular Vesicles (32).

### 2.3 Transmission electron microscopy

Transmission electron microscopy (TEM) was used to study the morphology of negatively stained EVs. First, a carbon copper grid (300 mesh) was placed on the purified EVs droplet (1:1000 diluted). Grid was incubated for 30 min at room temperature. Then, the copper grid was washed with PBS twice and dried. Finally, negative staining was done with 4% uranyl acetate. Morphologies of EVs were observed using a Zeiss EM 109T TEM with Gatan ES1000W digital camera.

### 2.4 Nanoparticle tracking analysis

The size distribution and concentration of nanoparticles were measured using ZetaView PMX-230 Twin Laser, Particle Metrix (NTA, Particle Metrix, Germany) with a 488 nm laser was used to analyze the EVs based on the characteristics of both light scattering and Brownian motion. EVs samples were diluted 1:10 with water free of nanoparticles and analyzed using SOP UC_EV_Scatter Eleven positions 30 frame rated video clips were recorded for every sample and ZetaView (version 8.05.16 SP7) software was used to analyze the data. The NTA is in the Instituto de Investigaciones en Microbiología y Parasitología Médica (IMPAM), Facultad de Medicina, Universidad de Buenos Aires, Argentina.

### 2.5 Tetraspanins analysis in extracellular vesicles

EVs tetraspanins expression were studied by EVs imaging cytometry analysis. It was adapted from Schürz et al. (33). Briefly, 5 x 10^8^ pEVs were incubated on microscopy slides at room temperature for 30 min to their adherence. To study CD9, CD63 and CD81 tetraspanins, once fixed with 4% paraformaldehyde for 10 min, the EVs were incubated 1 h with blocking solution (10% EVsdFCS in PBS), and incubated with primary antibody in blocking solution overnight at 4 °C into a humidity chamber. Then, slides were washed with PBS and incubated with conjugated secondary antibodies Alexa Fluor 488 or 594 (ThermoFisher Scientific) diluted 1:1000 in blocking solution. Finally, slides were washed with PBS and mounted for microscopy. Images, with at least 10 fields per well, were acquired with an EVOS M7000 (ThermoFisher Scientific Cat. AMF7000) and analyzed with the EVAnalyzer plugin (33) of FIJI software (NIH– ImageJ).

### 2.6 Western blot analysis

Lysates from explants were obtained with ice-cold modified RIPA buffer (50 mM Tris-HCl pH 7.4, 150 mM NaCl, 0.1% sodium dodecyl sulfate, 0.5% deoxycholate, 1% NP-40). Lysates were centrifuged at 10000 x g for 20min at 4 °C transferring the supernatants to a fresh tube. Laemmli buffer 4X was added to supernatant supplemented with 1% beta-mercaptoethanol. EVs final samples were directly mixed with Laemmli buffer 4X and heated at 95 °C for 5 min. Proteins samples were separated by 12% SDS-PAGE electrophoresis and transferred onto polyvinylidene difluoride membranes (GVS PVDF Cat. 1214429). Following this, the membranes were blocked in 3% bovine serum albumin dissolved in Tris-buffered saline (TBS) and incubated with primary antibodies overnight at 4 °C (Calnexin BD 610524 1:1000; Flotillin-1 BD 610821 1:1000). Fluorescent secondary anti-mouse antibody AzureSpectra 800 (Azure Biosystems) was employed, and blots were normalized by total line protein content using the TotalStain Q (PDVF) solution (Azure Biosystems Cat. AC2226). Images were acquired with a Sapphire Biomolecular Imager (Azure Biosystems) and quantified with FIJI software (NIH - ImageJ).

### 2.7 Sex-stratified reanalysis of previously published dataset on chorionic villi leukocytes

To assess potential sexual dimorphism in Hofbauer cells (HBCs), monocytes and macrophages associated to the placenta (PAMMs), we performed a *post hoc* sex-stratified reanalysis of the dataset of term placenta villi samples (34), segregating lean individuals by sex and re-evaluating phenotypic and functional parameters.

### 2.8 Flow cytometry

3 x10^5^ Mo were cultured without (-) or with 2–3 x 10^7^ EVs-derived from male or female, M-pEVs and F-pEVs, placentas in a 48 well flat-bottom polystyrene plates, in RPMI-160 medium (RPMI) 2% EVdFCS and after 20 h cells were recovered by Trypsin-EDTA (0.25%, Gibco) and stained for:

#### 2.8.1 Surface markers

with Fluorescein Isothiocyanate (FITC-), Phycoerytrin (PE-), Phycoerytrin-Cyanine 5 and 7 (PECy5 and 7-) or Allophycocyanin (APC-) conjugated mAbs directed to CD11b (BD Biosciences, Cat. No. 555389, RRID: AB_395790), CD11c (BioLegend, Cat. No. 301613, RRID: AB_493023), CD14 (BioLegend Cat. No. 325617, RRID: AB_830690), CD16 (Biolegend, Cat. No. 360705, RRID: AB_2616904), CD39/ENTPD1 (ectonucleoside triphosphate diphosphohydrolase 1. Biolegend Cat. No. 328205, RRID: AB_940423), CD86 (BD Biosciences, Cat. No. 550889, RRID: AB_396012), CD206/MRC1 (mannose receptor C-type 1, BD Biosciences, Cat. No. 550889, RRID: AB_398476).

#### 2.8.2 Metabolism probes

DCFH-DA (2’,7’-Dichlorodihydrofluorescein diacetate)-FITC (Cat. No D399) for total reactive oxygen species (ROS), BODIPY (4,4‐difluoro‐3a,4a‐diaza‐s‐indacene)-FL C12 (Cat.No. D3822) for long chain fatty acid uptake, BODIPY 493/503 (Cat. No. D3922) for lipid droplets accumulation, MitoTracker CMXRos (Cat. No. M46752) and MitoSpy Green (Biolegend Cat. No 424805) to assess mitochondrial membrane potential and mass, respectively. All probes were obtained from Thermo Fisher Scientific unless when is described other source.

Fifty thousand events were acquired in a FACS Aria II cytometer^®^ (Becton Dickinson) and results were analyzed using FlowJo software (http://www.flowjo.com/). Results were expressed as the percentage of the respective population and the quadrant was set using the autofluorescence control and were expressed as mean fluorescence intensity (M.F.I.) or double positive cell frequencies.

### 2.9 ELISA

Enzyme-Linked ImmunoSorbent Assay (ELISA) was performed to determine IL-1β, IL-6, IL-10 and CXCL8 levels in Mo/MA supernatant in absence or presence of pEVs using a commercial kit (BD Biosciences), according to the manufacturer’s recommendations and as previously described (35).

### 2.10 Lactate secretion

Lactate concentration in supernatants from Mo cultured for 20 h was measured with L-Lactate kit (Wiener Lab) according to the manufacturer’s instructions with minor modifications as previously described (17).

### 2.11 Reactive oxygen species production

After culturing, cells were stained with the specific fluorescence probe DCFH-DA (2’,7’-Dichlorodihydrofluorescein diacetate)-FITC as previously described (17). This probe was incubated for 10 min, washed and incubated with CD14 antibody to analyze double positive cells and MFI by flow cytometry.

### 2.12 Long chain fatty acids uptake

Long-chain fatty acid (LCFA) uptake was assessed using the fluorescent probe BODIPY-FL C12 (Molecular Probes– Life Technologies, CA, USA). BODIPY-FL C12 is a fluorescently labeled 12-carbon saturated fatty acid that functionally resembles an 18-carbon fatty acid (36). The probe was preincubated with 0.1% fatty acid free bovine serum albumin (FAF‐BSA, Sigma) for 30 min at 37 °C. Cells were washed twice with PBS and incubated with 5 μM BODIPY-FL C12 solution in serum‐free RPMI for 10 min at 37 °C, 5% CO2. Cells were washed with 0.2% BSA, resuspended in FACS solution and data was acquired as for glucose uptake assay. Results were expressed as the BodiPY FL C12 M.F.I. in CD14 positive cells.

### 2.13 Lipid droplets accumulation

Following two washes with PBS, cells were incubated to 2 μM BODIPY 493/503 in PBS for 15 min at 37 °C and 5% CO_2_. Cells were then washed with cold PBS, collected using 0.25% Trypsin-EDTA, and resuspended in FACS buffer prior to flow cytometry, as previously described. Results are presented as the mean fluorescence intensity (MFI) of BODIPY 493/503 in CD14 positive cells.

### 2.14 Analysis of membrane potential and mitochondrial mass

Cells were incubated with fluorescent probes MitoTracker CMXRos and MitoSpy Green to assess mitochondrial membrane potential and mass as previously described (27).

### 2.15 Efferocytosis assays

Phagocytosis of autologous apoptotic neutrophils (efferocytosis) in 3x10^5^ Mo/MA CD14 positive cells were performed as (24). Neutrophils were obtained after the Ficoll-Hypaque gradient and subsequent Dextran purification (37, 38). Apoptotic neutrophils were obtained after 20 h incubation in RPMI (spontaneous apoptosis) and stained with 3 µM/10^6^ cells of CFSE (Life Technologies, Buenos Aires) for 10 min in RPMI without FCS. Excess of CFSE was eliminated by serial washes with RPMI 10% FCS. The percentage of neutrophil apoptosis was higher than 50% as determined by annexin-propidium iodide staining and flow cytometry (24, 38).

For metabolic pathways inhibition, 10 mM 2-deoxy-d-glucose (2-DG), 100 nM rotenone (ROT) or 10 uM Etomoxir (ETO) were added to Mo/MA cultures 90 min before efferocytosis assay to inhibit glucose utilization, mitochondrial electron transport chain or, or fatty acid oxidation. Cells were incubated with apoptotic neutrophils in a 1:5 ratio and after 50 min of efferocytosis cells were collected, immunostained for CD14 and the percentage of CD14/CFSE double-positive cells was analyzed by flow cytometry as described above.

### 2.16 Statistical analysis

Statistical outliers were identified and excluded using the ROUT method (Q = 1%) prior to hypothesis testing. Data distribution was assessed for normality using the Shapiro–Wilk test. Comparisons between two groups were performed using two-tailed Mann–Whitney U tests or Wilcoxon matched-pairs signed-rank tests for nonparametric data, as appropriate. For comparisons involving multiple groups, ordinary one- or two-way ANOVA tests were used, followed by Holm–Šidák or Tukey’s multiple comparisons *post hoc* tests, as appropriate. For two-way ANOVA analyses with unequal sample sizes, Type III sums of squares were applied. Adjusted P values derived from multiple comparisons tests were used to control the family-wise error rate (FWER). Extracellular vesicle samples were evaluated and utilized individually per donor without pooling. In all quantitative figures, data are represented as individual scatter dot plots, where each dot represents the mean of an independent biological replicate with at least two different EVs/sex, to reflect biological variability. For imaging-based analyses, multiple fields of view were averaged per sample to obtain a single biological replicate for statistical analyses. Data are presented as mean ± SEM, and differences were considered statistically significant at P < 0.05.

## 3 Results

### 3.1 Sex-dependent differences in placental extracellular vesicles and their impact on monocyte phenotype

To investigate whether fetal sex influences placental extracellular vesicle properties and their effects on immune cells, placental extracellular vesicles (pEVs) were isolated from term placental explants from pregnancies carrying male or female neonates to evaluate potential sex-specific differences in vesicle properties and biological effects. pEVs were characterized according to the Minimal Information for Studies of Extracellular Vesicles (MISEV2023) guidelines from the International Society for Extracellular Vesicles (32).

**Figure 1:**
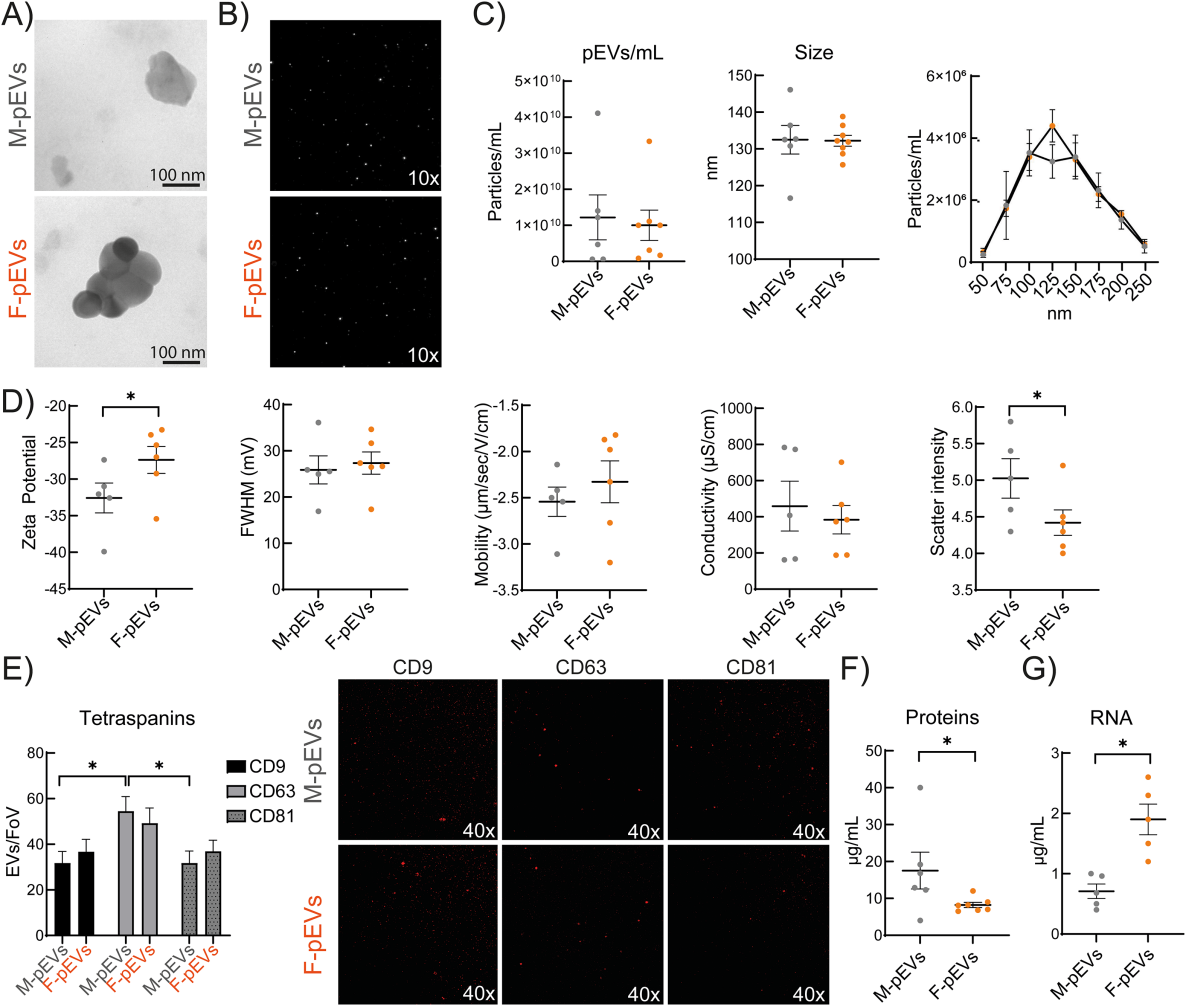
Placental extracellular vesicles display sex-specific physicochemical properties. Human placental villous explants from term pregnancies (male, M, n = 6; female, F, n = 7) were cultured to obtain placenta-derived extracellular vesicles (pEVs). **(A)** Representative transmission electron microscopy (TEM) micrographs from ≥3 independent experiments (85,000× magnification). **(B, C)** Nanoparticle tracking analysis (NTA) showing representative images (10× magnification), particle concentration, size, and distribution (gray: M-pEVs; orange: F-pEVs). **(D)** Physicochemical parameters including zeta potential, FWHM, mobility, conductivity, and scatter intensity. **(E)** Tetraspanin expression (CD9, CD63, and CD81) assessed by imaging flow cytometry (EVs/field of view; ≥20 fields/sample); representative images acquired at 40× magnification are shown. **(F)** Total protein content measured by microBCA assay and **(G)** RNA concentration measured by NanoDrop. Data are presented as mean ± SEM; each point represents an independent pEV preparation. Statistical analyses were performed using Mann–Whitney tests for panels **(C, D, F, G)**, and ordinary two-way ANOVA (Type III sum of squares) followed by Tukey’s multiple comparisons *post hoc* test for panel **(E)** Adjusted P values were used to account for multiple comparisons by controlling the family-wise error rate (FWER). *P < 0.05 was considered statistically significant.

Transmission electron microscopy confirmed the presence of heterogeneous populations of spherical nanosized vesicles with typical EV morphology in both groups (**Figure 1A**). Nanoparticle tracking analysis (NTA) showed similar particle concentrations per milliliter and size distributions between vesicles-derived from male (M-pEVs) and female (F-pEVs) placentas (**Figures 1B, C**). Integrated NTA analysis plotting particle size versus concentration confirmed overlapping distributions between the two populations of vesicles (**Figure 1C**).

Despite their similarities, analysis of physicochemical parameters revealed differences between the two populations of EVs. M-pEVs had a significantly lower zeta potential than F-pEVs and exhibited higher scatter intensity, indicating differences in vesicle composition or membrane organization (**Figure 1D**). No differences were observed in full width at half maximum (FWHM), mobility, or conductivity.

To further characterize nanovesicle surface markers, imaging cytometry was used to quantify the number of pEVs expressing the canonical tetraspanins CD9, CD63, and CD81. Quantification of EV-positive events per field of view (EV/FoV) revealed a comparable abundance of tetraspanin-positive vesicles in both preparations (**Figure 1E**). However, M-pEVs displayed relatively higher CD63 representation compared to CD9 and CD81, while F-pEVs exhibited a more balanced distribution of the three tetraspanins.

Remarkably, the µBCA assay revealed that M-pEVs had twice the protein concentrations of F-pEVs (**Figure 1F**), despite similar pEV purity evaluated by particle-to-protein ratios (**Supplementary Figure 1A**). To further validate the technical purity and rule out intracellular organelle contamination in our preparations according to MISEV criteria, we evaluated the presence of the endoplasmic reticulum marker Calnexin and the positive vesicle marker Flotillin-1 by Western Blot (**Supplementary Figures 1B, C**). While Calnexin was highly abundant in whole tissue lysates, it was completely undetectable in both M-pEV and F-pEV fractions. Concurrently, Flotillin-1 was robustly identified within the isolated pEV samples, confirming successful vesicle recovery free from structural cellular contamination. Finally, RNA content was measured by Nanodrop, revealing lower concentration in the M-pEVs preparation (**Figure 1G**). Together, these data indicate that, although M-pEVs and F-pEVs share similar concentrations and sizes, they exhibit distinct physicochemical properties and molecular cargo.

**Figure 2:**
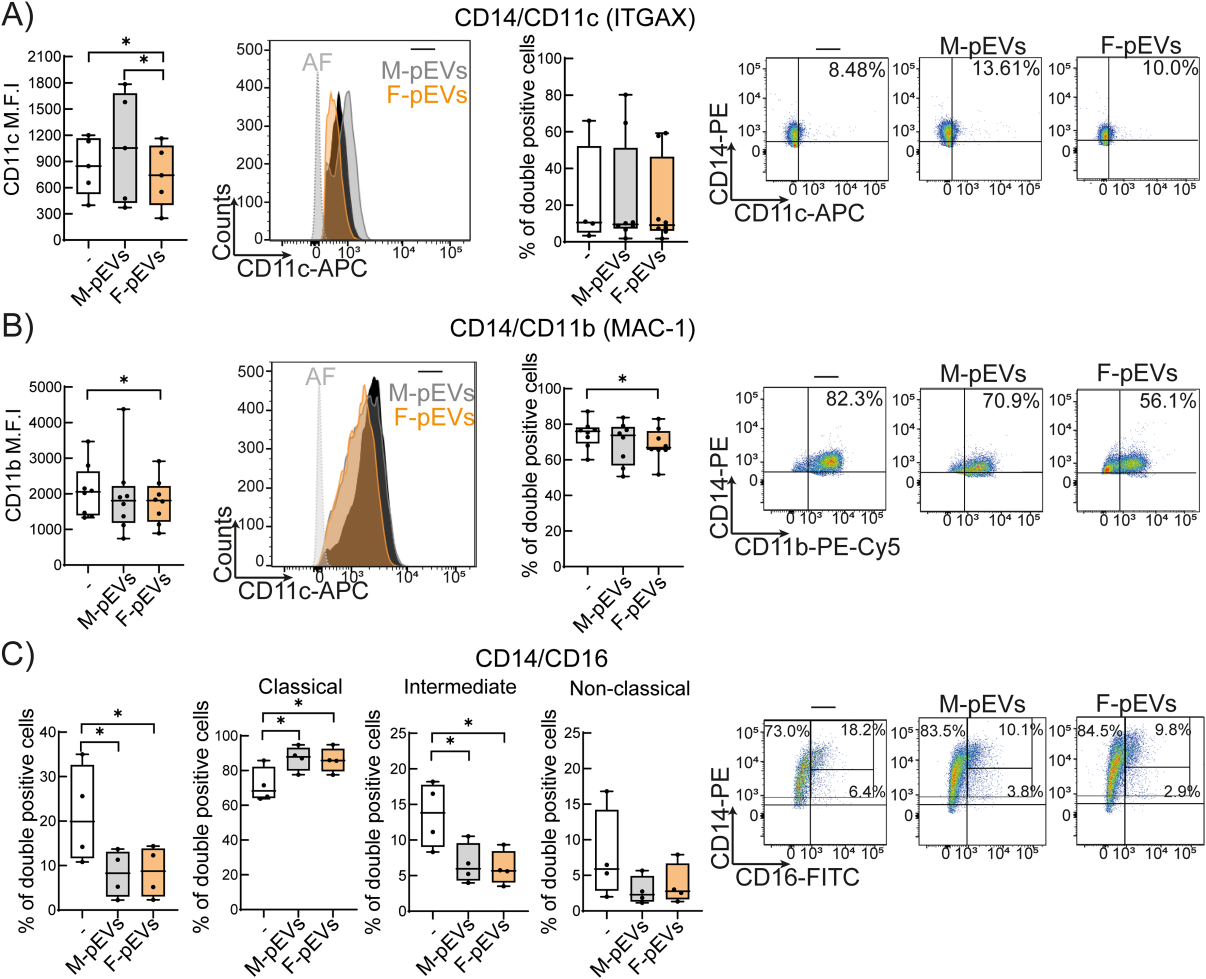
pEVs differentially shape monocyte adhesion markers and subset distribution according to fetal sex. Monocytes from non-pregnant donors were cultured o.n. in basal conditions (-) or with M-pEVs or F-pEVs and analyzed by flow cytometry for CD11c **(A)**, CD11b **(B)**, and CD16 expression and subset distribution **(C)**. The percentage (%) of double-positive cells and mean fluorescence intensity (M.F.I.) were evaluated. Representative histograms and dot plots of live cells are shown. Results are presented as mean ± SEM; each point represents an independents donor, and pEV conditions correspond to the mean of ≥ 2 independent pEV preparations. Gray: M-pEVs; Orange: F-pEVs. Statistical analyses were performed using ordinary one-way ANOVA followed by Tukey’s multiple comparisons *post hoc* test. Adjusted P values derived from Tukey’s multiple comparisons test were used to control the family-wise error rate (FWER). *P < 0.05 was considered statistically significant.

To explore whether these differences are associated with sex-specific immune profiles of monocyte (Mo), we next investigated whether pEVs could modulate their phenotype in a sex-dependent manner. Mo isolated from peripheral blood of non-pregnant donors were cultured overnight (o.n.), in the absence or presence of pEVs derived from male or female placentas. Exposure to F-pEVs significantly reduced the expression of two integrins associated with monocyte migration and inflammatory activation: CD11c (**Figure 2A**) and CD11b (**Figure 2B**), whereas M-pEVs did not significantly affect their expression.

Interestingly, both types of placental vesicles similarly reduced CD16 expression on monocytes. Analysis of monocyte subsets based on CD14 and CD16 expression revealed that both vesicle populations increased the proportion of classical monocytes while reducing the frequency of intermediate monocytes, with no effect on the non-classical subset (**Figure 2C**). These findings suggest that placental extracellular vesicles differentially modulate monocyte phenotype in a sex-dependent manner.

### 3.2 Placental extracellular vesicles induce distinct activation programs in circulating monocytes consistently with term placental leukocytes

**Figure 3:**
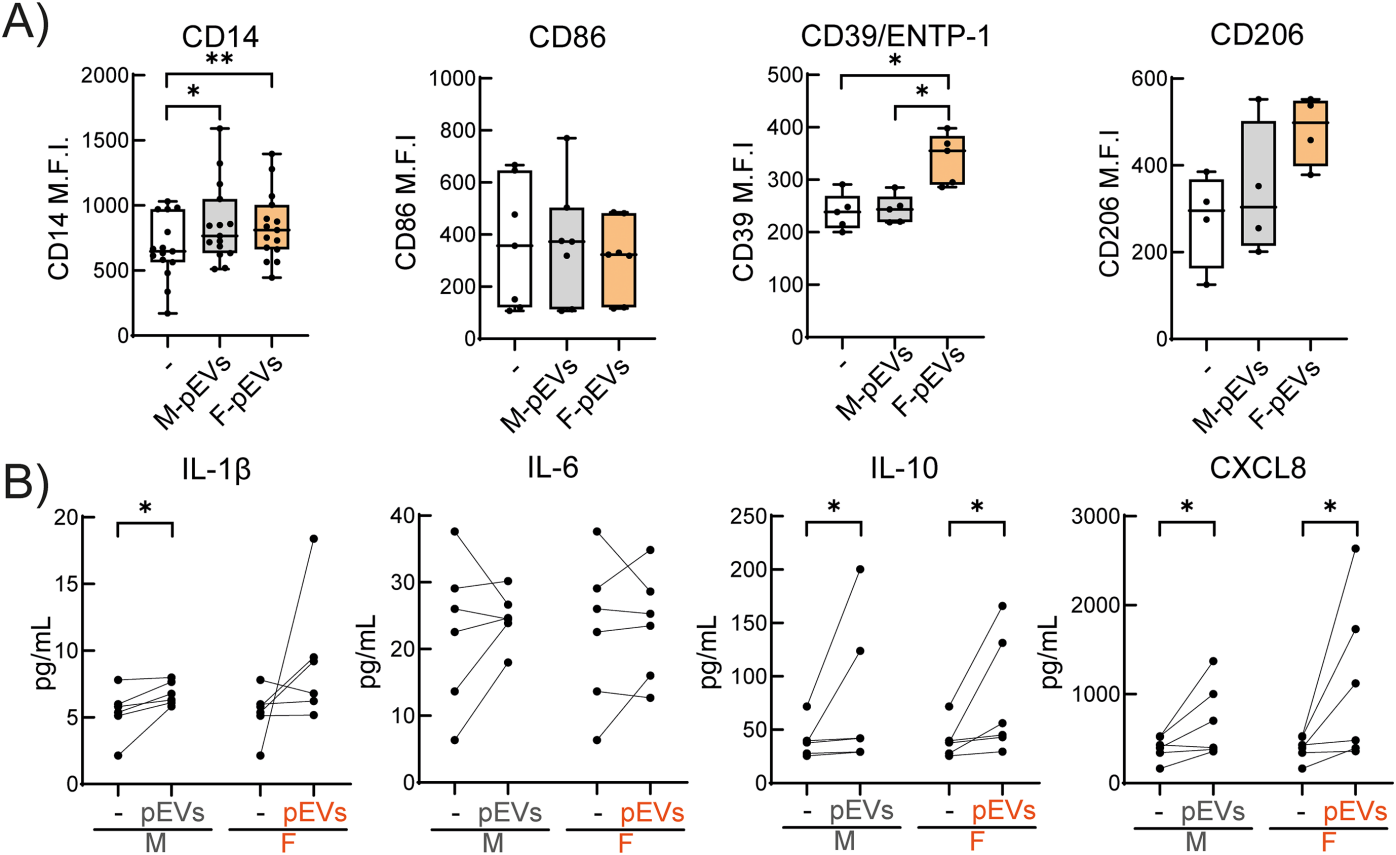
pEVs induce a distinct monocyte activation profile depending on fetal sex. Monocytes from non-pregnant donors were cultured o.n. in basal conditions (-) or in the presence of M-pEVs or F-pEVs and analyzed for: **(A)** Cell surface markers (CD14, CD86, CD39 and CD206) were evaluated by flow cytometry. **(B)** cytokine production (IL-1β, IL-6, IL-10 and CXCL8) was measured by ELISA. Results are expressed as mean ± SEM (MFI) and mean pg/mL; each point represents an independent donor, and pEV conditions correspond to the mean of ≥2 independent pEV preparations. Gray: M-pEVs; orange: F-pEVs. Statistical analyses were performed using ordinary one-way ANOVA followed by Holm–Šidák or Tukey’s multiple comparisons *post hoc* tests, as appropriate. Adjusted P values were used to control the family-wise error rate (FWER). *P<0.05 and **P<0.01 were considered statistically significant.

Based on the observed sex-specific differences in pEV properties and their effects on monocyte phenotype, we next evaluated whether these vesicles modulate the activation profile of circulating Mo. Mo from non-pregnant donors were cultured o.n. in the presence of M-pEVs or F-pEVs, and surface markers and cytokine production were assessed. Flow cytometry analysis revealed that M-pEVs significantly increased CD14 without changes in CD86 or anti-inflammatory markers expression (**Figure 3A**). In contrast, F-pEVs not only increased CD14 expression, but also induced a significant upregulation of CD39 (ENTPD1), with a trend towards increased CD206 expression.

Cytokine analysis revealed additional functional differences between the nanovesicle populations. Both M-pEVs and F-pEVs significantly increased the production of the anti-inflammatory cytokine IL-10, as well as the neutrophil-attracting chemokine CXCL8 (**Figure 3B**). However, only M-pEVs significantly increased IL-1β production, while IL-6 levels remained unchanged under all conditions.

Together, these data indicate that pEVs differentially modulate monocyte activation depending on fetal sex: F-pEVs favor a more regulatory phenotype, whereas M-pEVs induce a hybrid inflammatory-resolutive state.

To explore whether these sex-dependent effects observed in Mo treated with pEVs are consistent with leukocytes at the placental level, we performed *post hoc* sex-stratified analysis on single-cell RNA-seq/CITE-seq data of leukocytes isolated from chorionic villi from term placental tissues obtained from lean individual with uncomplicated pregnancies (34). The analytical workflow used is shown in **Supplementary Figure 2A**.

Comparing placental samples according to fetal sex revealed that macrophage populations from female placentas displayed reduced expression of molecules associated with classical inflammatory activation. Specifically, Hofbauer cells (HBCs) and placenta-associated maternal macrophages and monocytes (PAMMs) exhibited lower levels of CD62L, CD64, CD11c, and CD86 when derived from female placentas (**Supplementary Figure 2B**). In contrast, the anti-inflammatory marker CD163 remained largely unchanged across most macrophage populations. These differences were not accompanied by changes in phagocytic activity toward E. coli particles (**Supplementary Figure 2C**).

Reduced CD11c expression was particularly evident in PAMM1a and PAMM1b macrophages, which originate from circulating maternal monocytes (Mo).

These data strongly support that the sex-specific profiles observed in placental leukocytes could be partially achieved by pEVs.

### 3.3 Placental EVs derived from male or female fetuses induce distinct metabolic programs in monocytes

Considering that immune cell phenotype and function are closely linked to the metabolic status, we next investigated whether pEVs modulate monocyte metabolism in a sex-dependent manner.

**Figure 4:**
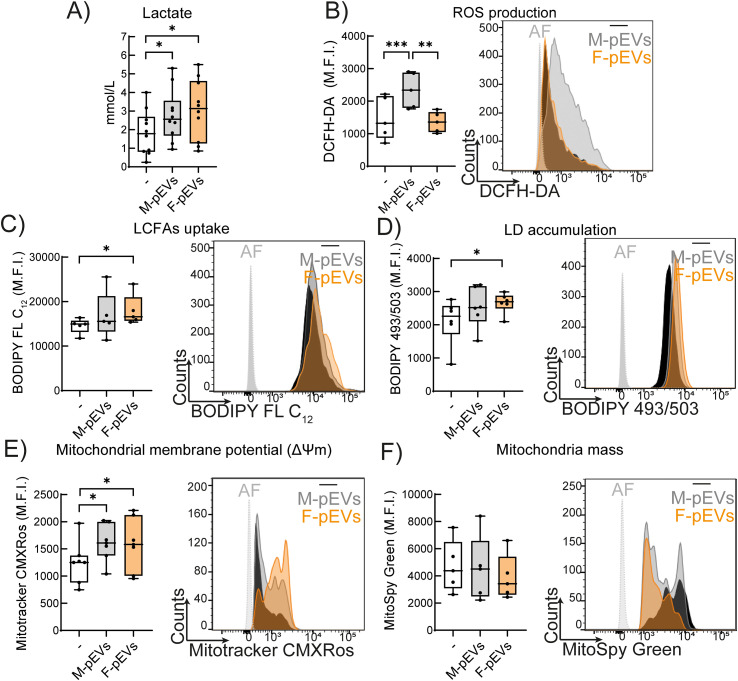
pEVs induce different metabolic rewiring in monoyctes depending on their origin. Monocytes cultured under basal conditions (-) or with M- or F-pEVs were analyzed for metabolic parameters. **(A)** Lactate production (mmol/L) was measured in supernatants by enzymatic assay. **(B)** Total ROS production, **(C)** long-chain fatty acid (LCFAs) uptake, **(D)** lipid droplet (LD) accumulation, **(E)** mitochondrial membrane potential, and **(F)** mitochondrial mass were assessed by flow cytometry. Representative histograms are shown. Data are presented as mean ± SEM (mmol/L, % of positive cells or MFI); each point represents an independent donor, and pEV conditions correspond to the mean of ≥2 independent pEV preparations. Statistical analyses were performed using RM-ordinary one-way ANOVA, followed by Tukey’s multiple comparisons *post hoc* tests. Adjusted P values were used to control the family-wise error rate (FWER). *P<0.05, **P<0.01 and ***P<0.001.

Analysis of lactate levels in supernatants of monocytes stimulated with placental EVs showed that both M-pEVs and F-pEVs significantly increased its production compared to unstimulated monocytes, consistent with enhanced glycolytic activity (**Figure 4A**). However, only Mo/MA exposed to M-pEVs exhibited increased production of total reactive oxygen species (ROS; **Figure 4B**). This finding highlights a sex-dependent difference in ROS modulation depending on the origin of the vesicles. Furthermore, evaluation of lipid metabolism-related parameters revealed changes specifically in Mo/MA exposed to F-pEVs compared to unstimulated and M-pEV-treated cells. This was characterized by increased uptake of long-chain fatty acids (LCFAs) (**Figure 4C**), together with significant accumulation of intracellular lipid droplets (**Figure 4D**).

Evaluation of mitochondrial parameters revealed that both EV populations increased mitochondrial membrane potential without affecting mitochondrial mass (**Figures 4E, F**), suggesting enhanced mitochondrial activity rather than mitochondrial biogenesis.

Together, these results indicate that placental EVs induce distinct metabolic rewiring in circulating monocytes depending on fetal sex. Both M-pEVs and F-pEVs increased lactate production, however, only M-pEVs were associated with increased ROS production, whereas F-pEVs selectively promoted lipid uptake and lipid droplet accumulation. In addition, both EV populations increased mitochondrial membrane potential without changes in mitochondrial mass.

### 3.4 Placental extracellular vesicles from male and female fetuses differentially engage metabolic pathways supporting efferocytosis

To evaluate the functional consequences of EV-induced metabolic rewiring in monocytes, we assessed their ability to perform efferocytosis of autologous apoptotic neutrophils (aPMN) in the presence or absence of specific metabolic inhibitors.

**Figure 5:**
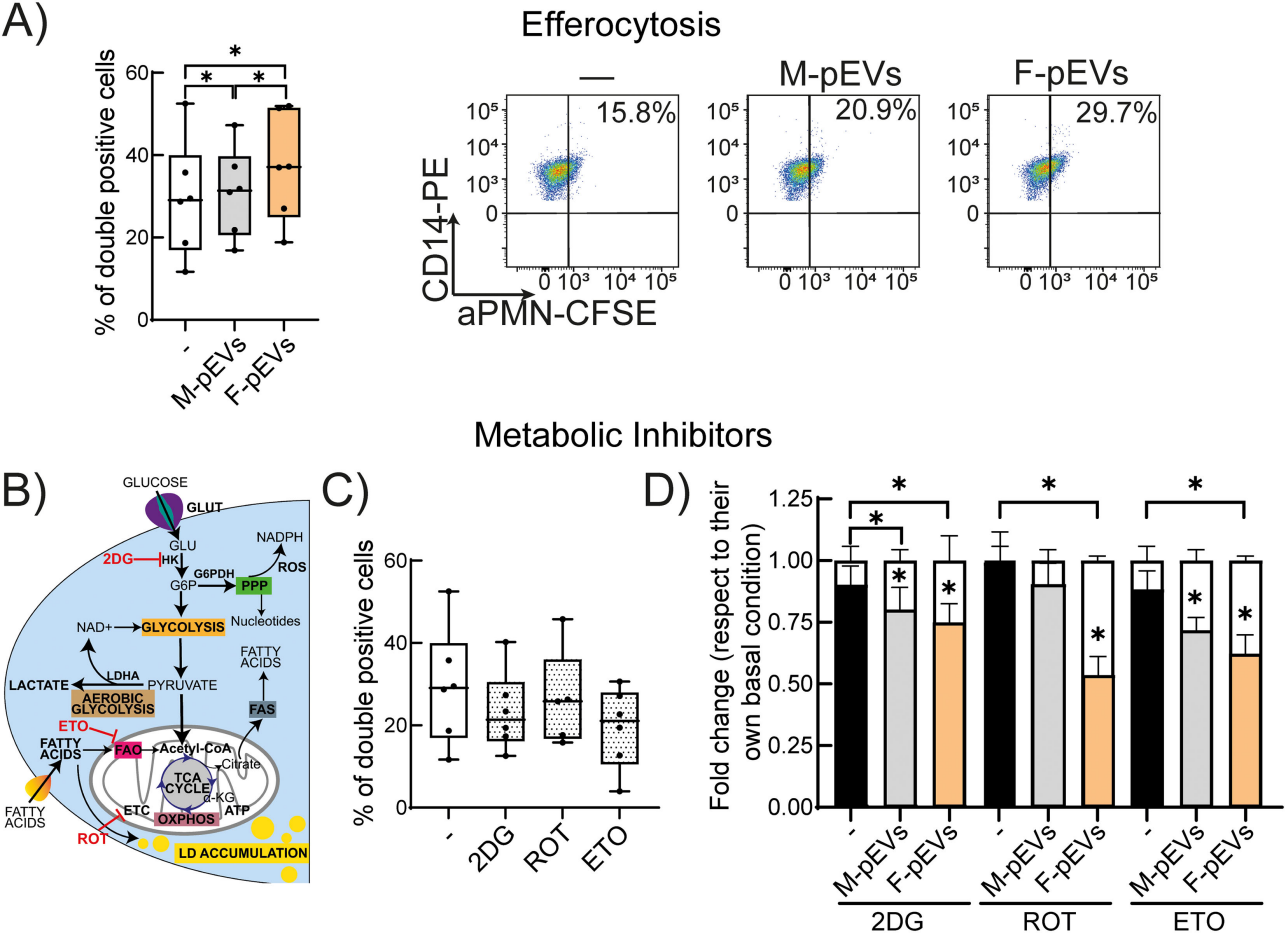
Efferocytosis induced by pEVs shows differential sensitivity to metabolic inhibition depending on vesicle origin. **(A)** Mo/MA cultured o.n. under basal conditions (-) or with pEVs were challenged with CFSE-labeled autologous apoptotic neutrophils (aNeu; 1:5 ratio, 1 h) and efferocytosis (CD14+CFSE+ cells) was assessed by flow cytometry. **(B)** Simplified schematic overview of metabolic pathways and pharmacological inhibitors used in the study. **(C)** Basal cells (-) were treated with metabolic inhibitors [2-deoxy glucose (2-DG;10 mM), Rotenone (ROT; 100 nM) or Etomoxir (ETO; 10 µM)] and subjected to the efferocytosis assay. Data are presented as mean ± SEM; each point represents an independent assay. Statistical analysis were performed using ordinary one-way ANOVA followed by Tukey’s multiple comparisons *post hoc* test for panels A and **(C)** *P< 0.05. D) Efferocytosis in control or pEVs treated cells in the presence of metabolic inhibitors. Data were normalized to their baseline condition (without inhibitors). White bar represents the baseline condition, whereas black, grey or orange bar represents inhibitor effects in (-), M-pEVS and F-pEVs conditions, respectively. Data are expressed as mean ± SEM. Statistical analysis was performed using ordinary two-way ANOVA (Type III sum of squares) followed by Holm–Šidák’s multiple comparisons *post hoc* test for panel **(D)** Adjusted P values derived from multiple comparisons tests were used to control the family-wise error rate (FWER). Asterisks indicate comparisons to the corresponding baseline condition (within bars) or to unstimulated conditions (above bars), as indicated in the figure. When it is inside of the bar it is against own basal condition but if the star is outside it is compared to Mo/MA without pEVs. Hexokinase (HK), glucose-6-phospate (G6P), G6P dehydrogenase (G6PDH), pentose phosphate pathway (PPP), reactive oxygen species (ROS), Lactate dehydrogenase A (LDHA), Tri-carboxylic acid (TCA), electron transport chain (ETC), oxidative phosphorylation (OXPHOS), fatty acid oxidation (FAO), fatty acid synthesis (FAS).

Both pEVs significantly enhanced the efferocytic capacity of monocytes compared to untreated controls, although the effect was significantly greater in Mo exposed to F-pEVs (**Figure 5A**).

To investigate the metabolic pathways supporting this process, Mo were treated with inhibitors targeting key metabolic pathways, including the glucose utilization inhibitor 2-deoxyglucose (2-DG), the mitochondrial complex I inhibitor rotenone (ROT), and the fatty acid oxidation inhibitor etomoxir (ETO). The targeted metabolic pathways are schematically represented in **Figure 5B**. These inhibitors did not significantly affect basal efferocytosis (**Figure 5C**). However, distinct metabolic dependencies were observed following Mo stimulation with placental EVs (**Figure 5D**). Treatment with the glycolysis inhibitor 2-DG significantly reduced efferocytosis in both M-pEV- and F-pEV-treated Mo compared to their respective conditions without the inhibitor. Moreover, in the presence of 2-DG, the increase in efferocytosis induced by pEVs relative to unstimulated Mo was no longer observed.

In contrast, inhibition of mitochondrial respiration and fatty acid oxidation revealed differences depending on the origin of the vesicles. While both pEV populations induced Mo partial dependence on fatty acid oxidation to support efferocytosis, inhibition of mitochondrial respiration produced a stronger reduction in efferocytosis in Mo exposed to F-pEVs. Specifically, while inhibition of fatty acid oxidation reduced efferocytosis in both M-pEV- and F-pEV-treated monocytes compared to their respective conditions without the inhibitor, this reduction reached statistical significance relative to unstimulated conditions only in F-pEV-treated monocytes. Moreover, inhibition of mitochondrial complex I with rotenone significatly reduced efferocytosis only in F-pEV-treated monocytes compared to both untreated and unstimulated conditions (**Figure 5D**).

Together, these results indicate a stronger sensitivity to metabolic inhibition in F-pEV-treated monocytes, particularly in response to mitochondrial perturbation.

## 4 Discussion

This study demonstrates that placental extracellular vesicles (pEVs) derived from term pregnancies differentially modulate circulating monocyte phenotype, metabolism, and function according to fetal sex. Despite similar size, concentration and tetraspanin expression, EVs-derived from male and female placentas differ in physicochemical properties, molecular composition. These differences were associated with distinct Mo activation states and immunometabolic programs.

M-pEVs displayed higher protein content and lower RNA levels, consistent with their increased scatter intensity and lower zeta potential compared to F-pEVs. These findings support the idea that EV composition and physicochemical properties vary not only with pregnancy and its complications (8, 39), but also according to fetal sex, potentially influencing colloidal stability (40).

Exposure to F-pEVs was associated with increased efferocytic capacity and metabolic features including lipid uptake, lipid droplet accumulation, and enhanced mitochondrial activity. In contrast, M-pEV-treated monocytes exhibited increased ROS production and lactate levels higher than basal condition, together with a distinct activation profile.

At the cellular level, F-pEVs-treated Mo showed reduced expression of the migration-associated integrins CD11b and CD11c. In contrast, CD14 and CD39 expression, as well as IL-10 production, were increased, A trend towards higher CD206 expression was also observed. These changes are consistent with a Mo activation profile associated with immune regulation and tissue homeostasis. CD39 expression on Mo/macrophages is linked to adenosine generation through ATP hydrolysis under physiological or inflammatory conditions (41, 42). Additionally, IL-10 and CD206 are characteristic of macrophage populations involved in immune regulation and tissue repair (25, 43, 44). In contrast, M-pEVs induced an activation profile characterized by increased CD14 expression, elevated IL-1β and CXCL8 production, and enhanced ROS generation, while maintaining IL-10 secretion. This profile is consistent with activation states described in circulating Mo during pregnancy, combining inflammatory mediators with regulatory signals (45–47).

Placenta-associated maternal macrophages (PAMM1b) are morphologically similar to circulating monocytes and contribute to inflammatory and phagocytic responses at the maternal–fetal interface (34, 48). These cells produce high levels of IL-1β, IL-6, and CXCL8 and exhibit greater phagocytic and microbicidal activity than PAMM1a cells. At term, they decrease migratory capacity (34). Reanalysis of placental datasets revealed that macrophage populations from female placentas exhibited reduced expression of markers associated with leukocyte trafficking, phagocytosis, and activation. Notably, these changes occurred without changes in phagocytic capacity (34). Importantly, similar patterns were observed in circulating monocytes exposed to F-pEVs, but not M-pEVs *in vitro*, suggesting that pEVs may participate in shaping these sex-specific activation profiles.

Metabolically, Mo exposed to M-pEVs exhibited increased glycolytic activity, elevated reactive oxygen species (ROS) production, and enhanced mitochondrial membrane potential without changes in mitochondrial mass. Glycolytic reprogramming is a hallmark of activated innate immune cells and supports biosynthetic and redox pathways required for inflammatory responses (49, 50). Such metabolic configurations are commonly associated with activated or stress-responsive monocyte states (51). These findings are in line with previous reports showing increased glycolytic dependency and altered oxidative metabolism in circulating monocytes during pregnancy (45).

In contrast, F-pEV-treated monocytes exhibited increased uptake of long-chain fatty acids, lipid droplet accumulation, and enhanced mitochondrial activity, despite an increase in lactate production and no increase in ROS production. This profile is consistent with previous studies describing the integration of glycolysis, fatty acid metabolism, and mitochondrial activity in circulating monocytes during pregnancy (26, 27).

Similar metabolic programs have been described in macrophages involved in tissue repair and resolution, which rely on fatty acid oxidation and mitochondrial respiration (43, 52, 53). In addition, lipid droplet accumulation may regulate lipid-derived mediators involved in the resolution of inflammation (54, 55).

These metabolic differences were accompanied by functional changes. Both EV populations enhanced efferocytosis, although F-pEVs induced a stronger effect. Glycolysis inhibition reduced efferocytosis in both conditions, indicating a shared requirement for glucose utilization. In contrast, inhibition of mitochondrial respiration and fatty acid oxidation had a greater impact on F-pEV-treated monocytes, revealing increased sensitivity to metabolic perturbation in this condition.

Beyond the local maternal-fetal interface, the uterine immune microenvironment exerts systemic influence through placental-derived extracellular vesicles that circulate in maternal bloodstream. Our data demonstrate that this systemic signal is not immunologically neutral: sex-associated differences in pEV composition may contribute to the differential reprogramming of circulating monocyte phenotype, metabolism, and function. Together, these findings suggest that fetal sex may influence maternal systemic immune tone through placental EV-mediated signaling. Given that male pregnancies are associated with a more pro-inflammatory maternal milieu and greater susceptibility to preeclampsia, the M-pEV-driven inflammatory-metabolic profile described here may reflect — and potentially contribute to — this sex-dependent immune asymmetry.

Two limitations of this study should be considered. First, extracellular vesicles were isolated from placental explants of comparable size and weight. All treatments were normalized by particle number to ensure a consistent vesicle-to-monocyte interaction ratio. This strategy enabled direct comparison of the biological effects of equivalent EV doses, while minimizing variability related to EVs abundance. Nevertheless, given the differences in physicochemical characteristics and molecular cargo between M-pEVs and F-pEVs, we cannot rule out the possibility that sex-specific EV composition contributed to the distinct immunometabolic responses observed.

Second, monocytes from non-pregnant donors were used as an experimental model. Pregnancy is associated with physiological monocyte priming, including metabolic reprogramming and changes in monocyte subset distribution. The use of non-pregnant donor cells therefore allowed us to specifically assess the direct effects of placental EVs, while minimizing the confounding influence of systemic pregnancy-related factors. Future studies using monocytes from pregnant individuals will be necessary to determine whether the immunometabolic responses identified here also occur *in vivo* and whether they are influenced by fetal sex.

To our knowledge, this is the first study to demonstrate sex-dependent differences in monocyte immunometabolic responses induced by term placental extracellular vesicles. These findings provide new insight into the mechanisms underlying maternal-fetal immune communication and support a potential role for placental EVs in shaping sex-specific maternal immune adaptation.

## Data Availability

The datasets presented in this study can be found in online repositories. The names of the repository/repositories and accession number(s) can be found in the article/**Supplementary Material**.

## Supplementary material

SUPPLEMENTARY FIGURE 1 Assessment of placental extracellular vesicles purity and protein marker profile. **(A)** To assess potential protein contamination, particles number informed by the NTA were normalized to the total protein concentration (ug) obtained by µBCA assay. Results are expressed as the mean ± SEM of the Particles/µg measured values, and each point represents an independent placental vesicle preparation. **(B)** Representative Western Blot membranes and relative densitometric quantification of the non-EV negative control marker Calnexin and the positive membrane-associated vesicle marker Flotillin-1 in whole placental tissue lysates (M- and F-) and isolated EV fractions (M-pEVs, F-pEVs). **(C)** Total protein stain of the membrane used as the loading control and reference baseline for densitometric normalization.

SUPPLEMENTARY FIGURE 2 Sex-specific phenotypic and functional markers in total chorionic villi leucocytes. **(A)** Workflow of publicly available scRNA/CITE-seq data from term placental leukocytes stratified according to the sex of the newborn. **(B)** surface marker expression (CD62L, CD64, CD11c, CD86, and CD163) and **(C)** E. coli phagocytosis in Hofbauer cells (HBCs, green), PAMM1a (red), PAMM1b (blue), and PAMM2 (purple) in male (M) and female (F) placentas. Data are presented as mean ± SEM of mean fluorescence intensity (MFI) for each marker and percentage of pHrodo-positive cells. Statistical analyses were performed using ordinary two-way ANOVA followed by Tukey's multiple comparisons post hoc test. Adjusted P values derived from Tukey's multiple comparisons test were used to control the family-wise error rate (FWER). P < 0.05 was considered statistically significant. *P < 0.05, **P < 0.01, and ***P < 0.001.
