# Supplementary figures and images for: Fetal sex shapes maternal immune adaptation: placental extracellular vesicles differentially reprogram the phenotype, metabolism, and function of circulating monocytes
Source: Front Immunol. 2026 Jul 17;17:1855111. doi: 10.3389/fimmu.2026.1855111 (PMC13424588; doi:10.3389/fimmu.2026.1855111)

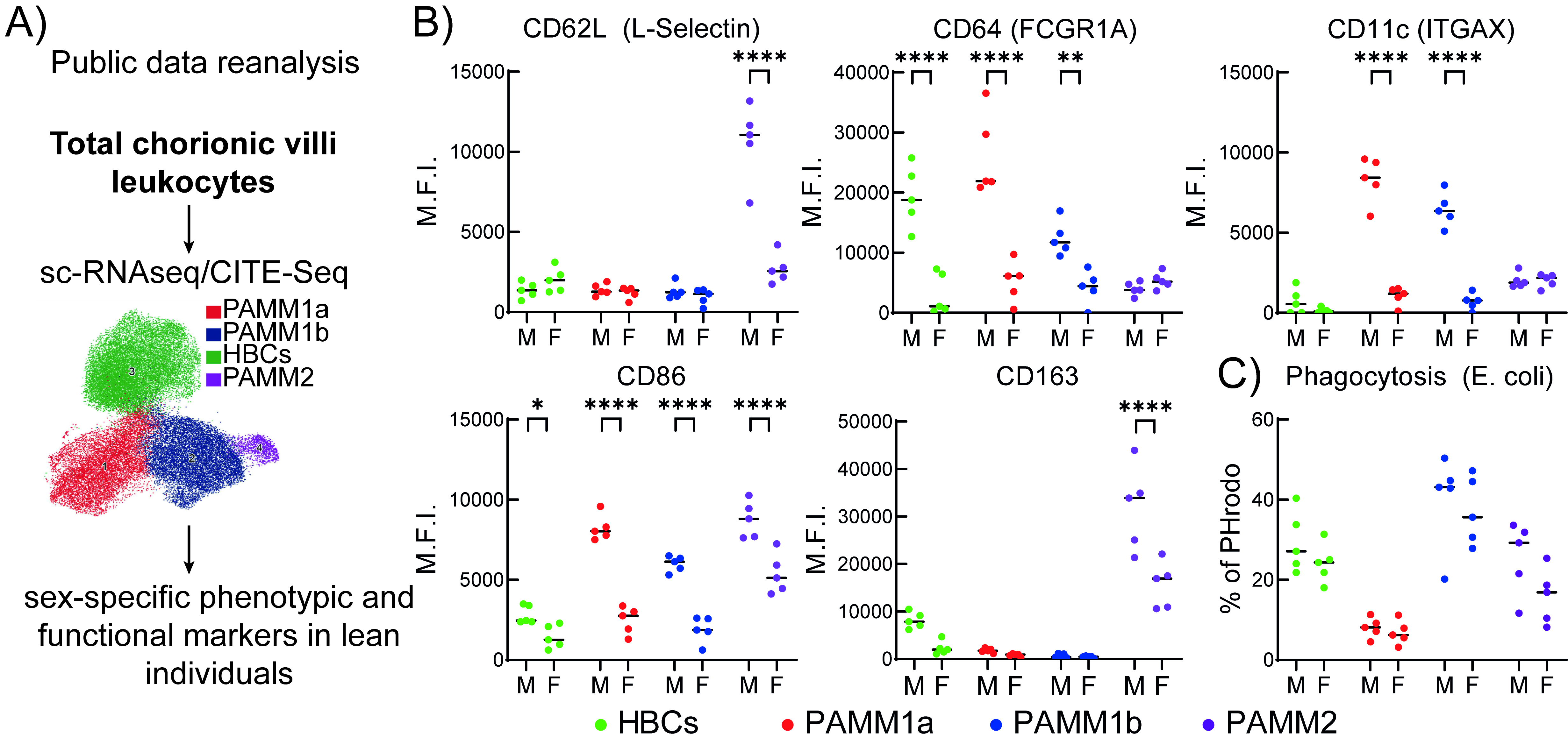

Supplement: SUPPLEMENTARY FIGURE 2 — Sex-specific phenotypic and functional markers in total chorionic villi leucocytes. (A) Workflow of publicly available scRNA/CITE-seq data from term placental leukocytes stratified according to the sex of the newborn. (B) surface marker expression (CD62L, CD64, CD11c, CD86, and CD163) and (C) E. coli phagocytosis in Hofbauer cells (HBCs, green), PAMM1a (red), PAMM1b (blue), and PAMM2 (purple) in male (M) and female (F) placentas. Data are presented as mean ± SEM of mean fluorescence intensity (MFI) for each marker and percentage of pHrodo-positive cells. Statistical analyses were performed using ordinary two-way ANOVA followed by Tukey's multiple comparisons post hoc test. Adjusted P values derived from Tukey's multiple comparisons test were used to control the family-wise error rate (FWER). P < 0.05 was considered statistically significant. *P < 0.05, **P < 0.01, and ***P < 0.001. [file Image2.tif]
